# Supplementary material for: Circular RNA circRNA-0039459 promotes the migration, invasion, and proliferation of liver cancer cells through the adsorption of miR-432
Source: Bioengineered. 2022 May 11;13(5):11810–21. doi: 10.1080/21655979.2022.2073129 (PMC9276028; doi:10.1080/21655979.2022.2073129)
Supplement: Supplemental Material [file KBIE_A_2073129_SM2357.zip › Revised manuscriptclean.docx]

**Circular RNA circRNA**-**0039459** **promotes the migration, invasion, and proliferation of liver cancer cells through the adsorption of miR-432**

Wenyong Zhou^1*^, Fengshuo Yang^2^

1. Department of General Surgery, Cangzhou Central Hospital, CangZhou, China
2. Department of Urology, Cangzhou People’s Hospital, CangZhou, China

*Corresponding to: Wenyong Zhou, Department of General Surgery, Cangzhou Central Hospital, No. 16 Xinhua West Road, Cangzhou City, 061001, Hebei Province, China. E-mail: zhouwenyong1101@163.com.

**Abstract**

This study aimed to investigate the molecular mechanism of circular RNA circ-0039459 and its effects on the apoptosis, proliferation, invasion, and migration of hepatocellular carcinoma cells. The expression of circ-0039459, miR-432, and synoviolin 1 (SYVN1) mRNA was determined using real-time quantitative reverse transcription PCR. Cell proliferation was detected by cell counting kit-8 assay, and the apoptosis rate was detected using flow cytometry. Cell migration and invasion were detected using Transwell assay. The expression of E-cadherin, N-cadherin, and vimentin was detected using western blot. The targeting relationship between circ-0039459 and miR-432 as well as that between miR-432 and SYVN1 were detected using the dual-luciferase reporter and RNA pull-down assays. We found that circ-0039459 and SYVN1 mRNA were highly expressed, whereas miR-432 was lowly expressed in hepatocellular carcinoma cells and tissues. After treatment with ribonuclease R or actinomycin D, the expression of linear RNA was reduced, whereas that of circular RNA was not significantly changed. circ-0039459 knockdown or miR-432 overexpression can inhibit cell proliferation, invasion, and migration and the expression of N-cadherin and vimentin proteins in carcinoma cells as well as promote apoptosis and increase the E-cadherin level. circ-0039459 targeted and regulated miR-432, which targeted and regulated SYVN1. The decreased miR-432 expression reversed the effects of circ-0039459 knockout in cancer cells. Furthermore, SYVN1 overexpression reversed the effect of miR-432 overexpression in hepatoma cells. Hence, circ-0039459 can affect the proliferation, apoptosis, migration, and invasion of hepatocellular carcinoma cells through the adsorption of miR-432, thereby regulating the expression of SYVN1.

**Keywords:** circ-0039459; miR-432; SYVN1; hepatocellular carcinoma; proliferation; metastasis; apoptosis

**Introduction**

Liver cancer is the sixth most common malignancy worldwide and ranks second among all cancer-related deaths. In East Asia, the incidence of liver cancer is the highest, followed by Southeast Asia and Africa ^[1, 2]^. Although the early diagnosis of liver cancer has progressed in recent years, many patients are already in the advanced stage at the time of diagnosis, and their cancer is often accompanied by distant metastases, which restricts treatment strategies and causes the incidence to rise ^[3]^. The key to improving treatment efficiency is to to find new molecular markers related to liver cancer and discover their mechanism of action.

Circular RNA (circRNA) is an endogenous non-coding RNA produced by the reverse splicing of introns, exons, or intergenic regions. Extensive research has shown that circRNA is involved in the development of various diseases ^[4]^. Furthermore, circRNA has a more stable structure than linear RNA; hence, it is better suited as a molecular marker of disease ^[5]^. CircRNA plays an important role in regulating cancer cell proliferation, apoptosis, migration, invasion, blood vessel production, and many signaling pathways. In addition, circRNA plays a biological role by regulating gene expression. It can directly regulate gene expression and indirectly regulate downstream gene expression through regulating microgene regulatory elements, such as microRNAs (miRNAs) and RNA-binding proteins ^[6]^.

miRNA can regulate the expression of the target gene by degrading its transcript or inhibiting its translation by complementary binding with the 3'-untranslated regions (UTR) of the target gene mRNA. ^[13]^ miRNA can regulate the proliferation, migration, apoptosis, differentiation, and other cell biological processes of tumor cells ^[15]^. Studies have reported that miR-432 can inhibit the growth of liver cancer ^[16]^, osteosarcoma ^[17]^, glioblastoma multiforme ^[18]^, and other tumors. Synoviolin 1 (SYVN1), an E3 ubiquitin ligase involved in endoplasmic reticulum-related degradation, can regulate endoplasmic reticulum stress, chronic inflammation, blood vessel growth, oxidative stress, cell apoptosis, and other physiological processes ^[20,21]^. A study has indicated that SYVN1 is related to the growth and metastasis of liver cancer ^[22]^. However, the relationship between circ-0039459, miR-432 and SYVN1 remains unknown.

We used the R software to analyze the GSE155949 dataset from the Gene Expression Omnibus (GEO) database. Next, we screened the differentially expressed circRNAs in liver cancer and found that circ-0039459 showed the most significant difference. Therefore, we chose circ-0039459 as the research object and aimed to discover the regulatory circ-0039459 on liver cancer and the downstream regulatory mechanism. We considered that circ_0039459 regulates biological behaviors including proliferation, migration, invasion, and apoptosis by modulating miR-432/SYVN1 axis. This study will provide a new approach for liver cancer treatment.

**Materials and Methods**

**Sample collection**

Samples of cancer and paracancerous tissues were collected from 57 patients with liver cancer undergoing resection in Cangzhou People’s Hospital, including 40 males and 17 females aged 29 to 79 years old with an average age of 53.9 years. No patient underwent chemotherapy or radiotherapy before surgery, and all patients agreed to participate in the study, and written informed consent was collected from each patient. The study was approved by the Ethics Committee of Cangzhou People’s Hospital (No.[2021]04-26-04).

**Main reagents**

The normal liver epithelial cell line THLE-3 and hepatocellular carcinoma cell line Hep3B, and Huh7 were obtained from ATCC (Manassas, VA, USA); Dulbecco's Modified Eagle Medium (DMEM) and fetal calf serum, from Gibco (Grand Island, NY, USA); LipofectamineTM 2000 kit, TRIzol reagent, RNA reverse transcription kit, and PCR, from Invitrogen (Carlsbad, CA, USA); and PCR primers and bicinchoninic acid (BCA) protein detection kit, from Shanghai Shenggong Bioengineering Co., Ltd., (Shanghai, China). In addition, CCK-8 reagent was purchased from Sigma-Aldrich (St. Louis, MO, USA); crystal violet stain, from Zhongshan Golden Bridge Biotechnology Co., Ltd. (Beijing, China); Annexin V-Fluorescein Isothiocyanate (FITC)/Propidium Iodide (PI) Apoptosis Kit, from the Shanghai Meiji Biomedical Technology Co., Ltd. (Shanghai, China); Matrigel, from Sigma-Aldrich (St. Louis, MO, USA); and Transwell chamber; from the Shanghai Yanhui Biotechnology Co., Ltd., (Shanghai, China). E-cadherin, N-cadherin, and vimentin antibodies were acquired from the Santa Cruz Co., (Santa Cruz, CA, USA). pmiR-GLO luciferase reporter vector: Promega Corporation (Madison, WI, USA), dual-luciferase activity detection kits were obtained from the Shanghai Biyuntian Biotechnology Co., Ltd (Shanghai, China). Streptavidin magnetic beads were purchased from New England Biolabs (Beverly, MA, USA).

**Cell culture and transfection**

The THLE-3, Hep3B, and Huh7 cells were cultured in DMEM containing 10% fetal bovine serum in a 37 °C, 5% CO_2_ saturated humidity incubator. Hep3B and Huh7 cells were collected in the logarithmic growth phase, and the LipofectamineTM 2000 kit instructions were followed to compare circ-0039459 small interfering RNAs (si-circ-0039459 1#: TTTGACTTGACTTGCCGCGCT, si-circ-0039459 2#: AACAGCTTTGACTTGACTTGC). In addition, the SYVN1 overexpression plasmid (oe-SYVN1) and negative control (oe-nc) were transfected into the Hep3B and Huh7 cells.

**Real-time quantitative reverse transcription PCR (qRT-PCR)**

Total RNA from liver cancer tissues or cells was extracted using the TRIzol method. The concentration and purity were determined, and an RNA reverse transcription kit was used to transcribe RNA to cDNA. Finally, using cDNA as the template and U6 as the internal reference for PCR amplification, the relative quantitative analysis of the expression of circ-0039459, miR-432, and SYVN1 was performed on ABI 7500 real-time PCR system using the 2^-△△Ct^ method. The sequences of the primers were listed as follows: circ_0039459: F 5′-CTGACCGTGACCGTTTGCTA-3′, R 5′-ATCCATGGCGAGCTGAAGAG-3′, miR-432: F 5′-CGACGCGTACTCAAACACTTCGGACATGG-3′, R 5′-CCCAAGCTTCAAAGAGCAACAGAGAGTAGCA-3′, SYVN1: F 5′-CTTCGTCAGCCACGCTTATC-3′, R 5′-CCACGGAGTGCAGCACATAC-3′, U6 F 5′-ATTGGAACGATACAGAGAAGATT-3′, R 5′-GGAACGCTTCACGAATTTG-3′, GAPDH F 5′-AACGTGTCAGTGGTGGACCTG-3′, R 5′-AGTGGGTGTCGCTGTTGAAGT-3′.

**Cell counting kit-8 (CCK-8) assay**

Hep3B and Huh7 cells were inoculated in a 96-well plate, and 100 μL of CCK-8 working solution was added at 12, 24, 48, and 72 h ^[15]^. The cells were then incubated for 1 h at 37 °C and under 5% CO_2_ and measurements were taken using a microplate reader. The optical density value of the well at 490 nm indicated the cell proliferation level.

**Flow cytometry to detect the cell apoptosis rate**

Flow cytometry was conducted as previously described ^[15]^. Hep3B and Huh7 cells were seeded into a 6-well plate and were cultured in DMEM and digested using trypsin. Next, the cells were washed with phosphate-buffered saline (PBS) and centrifuged. The cells were collected, and 500 μL of binding buffer was added, followed by 5 μL each of annexin V-FITC and PI. Finally, the solution was mixed well and placed in the flow cytometer for 1 h.

**Transwell assay**

Transwell assay was performed as previously described ^[16]^. Hep3B and Huh7 cells were seeded and cultured at 37 °C. In the migration experiment, the 100 μL cell suspension was added to the Transwell chamber, and 500 μL of DMEM containing fetal calf serum was added to the lower Transwell chamber. The cells were cultured at 37 °C in a 5% CO_2_ incubator for 48 h and then removed and wiped with a cotton swab. Next, they were stained with crystal violet for 15 min and washed with PBS. The cells were then observed through a filter membrane under an inverted microscope, and the number of migrated cells was determined by counting five fields and taking the average. In the invasion experiment, the upper Transwell chamber was coated with 100 μL of Matrigel (dilution ratio 1:8) diluted with DMEM. The remaining steps were the same as those for the migration experiment.

**Western blotting**

The culture medium of the Hep3B and Huh7 cells cultured for 48 h was discarded, RIPA lysate was added to extract the total cell protein, and sodium dodecyl sulfate (SDS)-polyacrylamide gel electrophoresis was used to separate the protein. The separated protein gel was then transferred to a polyvinylidene fluoride (PVDF) membrane at 25°C. After using 5% skim milk for blocking, primary antibody diluent was added (E-cadherin 1:2000, N-cadherin 1:2000, and vimentin 1:2000). After washing with PBS, the gel was incubated at room temperature with secondary antibody dilution solution (1:5000) for 1 h and washed again with PBS. Finally, electrochemiluminescence was added dropwise, and the gel was exposed to X-ray film in a dark room, which was then developed and fixed. GAPDH was selected as the internal reference, and ImageJ image analysis software was used to determine the integrated absorbance of the protein band.

**Dual-luciferase reporter assay**

Bioinformatics analysis showed that the 3′-untranslated region (3′UTR) of circ-0039459 and SYVN1 mRNA had possible complementary nucleotide sequences to miR-432. The wild-type (circ-0039459-wt) and mutant (circ-0039459-mut) circ-0039459 pmiR-GLO luciferase reporter vector as well as the synthetic wild-type (SYVN1-wt-3′UTR) and mutant (SYVN1-mut-3′UTR) SYVN1 3′-UTR luciferase reporter vector were co-transfected with negative control (nc mimic) or miR-432 mimic (mimic) into Huh7 and Hep3B cells. Afterward, these cells were cultured for 48 h at 37 °C and 5% CO_2_.

**RNA pull-down assay**

RNA pull-down assay was performed as previously described ^[16]^. After 48 h of transfection, the cells were collected, washed with PBS, and incubated with cell lysis buffer, M-280 streptavidin magnetic beads pre-coated with RNase-free, and yeast tRNA for 3 h at 4 °C. Next, the cells were washed with cold lysis buffer, followed by washing three times with low-salt buffer and once with a high-salt buffer. Finally, the bound RNA was purified with TRIzol, and the expression levels of circ-0039459 and SYVN1 mRNA were detected by qRT-PCR.

**Statistical analysis**

Data were analyzed using the SPSS26.0 statistical software to obtain measurements and counts (mean ± standard deviation; total number, %) and comparisons between cohorts were made using the student’s t test. In addition, comparisons between multiple groups were made using the one-way analysis of variance. The difference was statistically significant when P < 0.05.

**Results**

In the current study, we explore the role of circ_0039459 in liver cancer and the underlying mechanisms. We detected the biological behaviors including cell proliferation, apoptosis, migration, invasion, and EMT. We found circ_0039459 was upregulated in liver cancer tissues and cells, which knockdown inhibited cell metastasis and facilitates apoptosis via regulating the miR-432/SYVN1 axis.

**Levels of circ-0039459 in liver cancer tissue and cells**

Using R software to analyze the differentially expressed circRNAs of liver cancer from the GEO dataset GSE155949, the seven circRNAs with the most noticeable differences were selected, and qRT-PCR analysis showed that their expression levels in Hep3B and Huh7 cells were higher than those in the normal liver epithelial cells THLE-3. The expression of circ-0039459 was the most apparent. In addition, the circ-0039459 level in cancer tissues was higher than that in adjacent tissues. After ribonuclease R or actinomycin D treatment, the linear RNA level reduced significantly, whereas the circRNA level did not significantly change, as shown in Figure 1.

**Effect of interference with the expression of circ-0039459 on apoptosis, migration, invasion, proliferation, and epithelial-mesenchymal transition (EMT) in liver cancer cells**

CCK-8 assay showed that interference with the expression of circ-0039459 significantly inhibited the proliferation of Huh7 and Hep3B cells. Furthermore, flow cytometry showed that interference with the circ-0039459 levels significantly promoted cell apoptosis. The Transwell assay showed that interference with the expression of circ-0039459 significantly inhibited the migration and invasion of Hep3B and Huh7 cells. Moreover, western blot analysis showed that the expression of circ-0039459 significantly inhibits that of E-cadherin and vimentin in Hep3B and Huh7 cells and promotes N-cadherin expression, as shown in Figure 2.

**Targeting relationship between circ-0039459 and miR-432**

Bioinformatics analysis showed that circ-0039459 has potential binding sites for miR-432. The dual-luciferase reporter assay for miR-432 mimic co-transfected with circ-0039459-wt luciferase reporter showed that the relative luciferase activity of the cells was significantly reduced. In contrast, the cells having miR-432 mimic co-transfected with circ-0039459-mut luciferase reporter vector showed no significant change in relative luciferase activity. Furthermore, the RNA pull-down experiment showed that miR-432 and circ-0039459 could specifically bind. The qRT-PCR analysis showed that interference with circ-0039459 expression could change the miR-432 expression; miR- 432 levels in Hep3B and Huh7 cells were significantly lower than those in THLE-3. Moreover, in liver cancer, the miR-432 expression in cancer tissues is lower than that in adjacent tissues, as shown in Figure 3.

**Inhibition of miR-432 expression reverses the effect of interference of circ-0039459 expression on the proliferation, apoptosis, migration, invasion, and EMT of liver cancer cells**

qRT-PCR was used to verify successful transfection and interference. The circ-0039459 expression significantly inhibited Hep3B and Huh7 cell migration, proliferation, and invasion; N-cadherin and vimentin expression significantly promoted cell apoptosis; and E-cadherin expression inhibited the miR-432 expression and reversed the interference with circ-0039459 expression. Figure 4 shows the effects of hepatocellular carcinoma cell apoptosis, proliferation, migration, and invasion as well as E-cadherin, N-cadherin, and vimentin expression.

**Targeting relationship between miR-432 and SYVN1**

Bioinformatics analysis showed that miR-432 has a potential binding site with the 3′UTR of SYVN1 mRNA. The dual-luciferase reporter assay for miR-432 mimic co-transfected with SYVN1-wt-3′UTR luciferase reporter vector showed that the luciferase activity in the cells was reduced. In contrast, the cells having miR-432 mimic co-transfected with SYVN1-mut-3′UTR luciferase reporter showed no significant change in relative luciferase activity. Furthermore, the RNA pull-down assay showed that miR-432 and SYVN1 could specifically bind, and qRT-PCR showed that it interfered with circ-0039459 expression. The si-circ_0039459 can inhibit SYVN1 mRNA, and inhibiting the miR-432 expression can reverse the inhibitory effect of interference with circ-0039459 expression on SYVN1 mRNA. The expression of SYVN1 mRNA in Hep3B and Huh7 cells was significantly higher than that in THLE-3. Furthermore, the expression of SYVN1 in liver cancer tissues was significantly higher than that in adjacent tissues, as shown in Figure 5.

**SYVN1 overexpression reverses the effects of miR-432 overexpression on the apoptosis, migration, invasion, proliferation, and EMT of liver cancer cells**

Hep3B and Huh7 cells were transfected with oe-SYVN1 and oe-nc, and qRT-PCR was used to verify the successful transfection. miR-432 overexpression inhibited the migration, proliferation, and invasion of Hep3B and Huh7 cells, and N-cadherin and vimentin expression significantly promoted cell apoptosis and E-cadherin expression. SYVN1 overexpression reverses miR-432 overexpression on the proliferation, migration, apoptosis, invasion of liver cancer cells and protein expression of E-cadherin, N-cadherin, and Vimentin, as shown in Figure 6.

**Discussion**

The majority of human RNA transcripts are non-coding RNAs, including circRNA, miRNA, and lncRNA, that do not encode proteins. Non-coding RNAs play an important role in regulating cell physiology and gene expression. CircRNA is closely related to the malignant biological behavior of cancer. For example, circRNA_0000285 can promote the proliferation and metastasis of cervical cancer cells by upregulating FUS ^[17]^, and circRNA_100876 can increase the growth of colorectal cancer cells by inhibiting the miR-516b expression ^[18]^. Furthermore, circRNA_001937 expression in skin squamous cell carcinoma is increased, and silencing its expression may inhibit the progression of this disease by regulating the miRNA-597-3p/FOSL2 pathway ^[19]^. In the present study, preliminary bioinformatics analysis and qRT-PCR found high expression of circ-0039459 in liver cancer tissues and cells. To clarify the effect of circ-0039459 on liver cancer, we studied its effects on the proliferation, apoptosis, migration, invasion, and EMT of liver cancer cell lines Hep3B and Huh7.

Tumors occurrence is related to progression and abnormal cell apoptosis, proliferation, migration, and invasion ^[20]^. Tumor cells often have the characteristics of excessive migration, invasion, and proliferation as well as a decreased apoptosis rate. Furthermore, tumor metastasis is regulated by EMT. During the EMT process, epithelial cells gradually transform into mesenchymal cells that cause cell migration and invasion ^[21]^. EMT is characterized by reduced E-cadherin and mesenchymal marker levels as well as increased N-cadherin and vimentin expression ^[22]^. We found that interference with circ-0039459 expression can inhibit Hep3B and Huh7 cell proliferation, migration, and invasion as well as N-cadherin and vimentin expression and promote cell apoptosis and E-cadherin expression. This indicates that interference with circ-0039459 expression can inhibit the progression of liver cancer.

Circular RNA can act as competitive endogenous RNAs (ceRNAs) that regulating miRNA expression ^[23]^. miRNA can regulate cell biological processes of tumor cells including proliferation, migration, apoptosis, and differentiation ^[8]^. MiR-432 is commonly act as a tumor suppressor. MiR-432 was the target of multiple circRNAs, such as circ-0021093, circ-100984, and circ-0132817, to regulate the progression of cancers ^[24-26]^. However, the interaction between miR-432 and circ-0039349 remains unknown. In the present study, using bioinformatics analysis, a dual-luciferase reporter assay, and an RNA pull-down experiment, we found that circ-0039459 has a targeting relationship with miR-432, and qRT-PCR testing further showed that circ-0039459 can target and regulate miR-432 expression, resulting in its low expression in liver cancer tissues and cells. Further analysis found that miR-432 overexpression can inhibit hepatocarcinoma cell proliferation, migration, and invasion as well as N-cadherin and vimentin expression. It can also promote cell apoptosis and E-cadherin expression and reverse the interference with circ-0039459 expression. The effects on the apoptosis, proliferation, migration, invasion, and EMT of liver tumor cells indicate that miR-432 can inhibit the malignant process of liver cancer, which is consistent with the results of previous studies ^[27]^. The findings suggested that circ-0039349 facilitates the progression of liver cancer by targeting miR-432.

MiRNAs regulates gene expression by base-pairing to the mRNA 3'-UTR ^[13]^. Dysregulation of SYVN1 is associated with the biological behaviors of breast cancer ^[28]^, lung cancer ^[29]^, and colon cancer ^[30]^. SYVN1 is involved in the progression of liver cancer ^[14]^.^.^ The present study found that miR-432 has a targeting relationship with SYVN1, and circ-0039459 regulates the expression of SYVN1 through miR-432. SYVN1 is highly expressed in liver cancer tissues and cells. Further analysis found that SYVN1 overexpression can reverse the effects of miR-432 overexpression on apoptosis, migration, proliferation, invasion, and EMT of liver cancer cells, consistent with previous studies ^[31,32]^. The findings indicating that miR-432 may exert a tumor suppressor effect by regulating SYVN1.

The limitation of this study was that we were unable to translate the results of in vitro study into in vivo study. We will conduct in vivo and even clinical studies on the role of circ_0039459 in liver cancer in our future work.

**Conclusion**

In summary, circ-0039459 plays a carcinogenic effect in liver cancer, and circ-0039459 targets miR-432 to regulate the expression of SYVN1, which may be the mechanism by which circ-0039459 affects the apoptosis, proliferation, invasion, migration, and EMT process of liver tumor cells.

**Acknowledgments**

Not applicable.

**Disclosure of interest**

No conflict of interest exits in the submission of this manuscript, and manuscript is approved by all authors for publication.

**Funding details**

Not applicable.

**Data availability statement**

The datasets used and analyzed during the current study are available from the corresponding author on reasonable request.

**References**

1. Ye Y, Xu Y, Lai Y, et al. Long non-coding RNA cox-2 prevents immune evasion and metastasis of hepatocellular carcinoma by altering M1/M2 macrophage polarization[J]. J Cell Biochem, 2018, 119(3):2951-2963.
2. Lei, Huang, Xinyu, et al. Long non-coding RNA linc-ITGB1 promotes cell proliferation, migration and invasion in human hepatoma carcinoma by upregulating ROCK1.[J]. Bioscience reports, 2018, 38(5):BSR20181289.
3. Li B, Mao R, Liu C, et al. LncRNA FAL1 promotes cell proliferation and migration by acting as a CeRNA of miR-1236 in hepatocellular carcinoma cells[J]. Life Sci, 2018, 197:122-129.
4. Su Y, Lv X, Yin W, et al. CircRNA Cdr1as functions as a competitive endogenous RNA to promote hepatocellular carcinoma progression[J]. Aging, 2019, 11(19):8183-8203.
5. Xu X, Zhang J, Tian Y, et al. CircRNA inhibits DNA damage repair by interacting with host gene[J]. Mol Cancer, 2020, 19(1):128.
6. Arnaiz E, Sole C, Manterola L, et al. CircRNAs and cancer: Biomarkers and master regulators[J]. Semin Cancer Biol, 2019, 58:90-99.
7. Zhou Z, Ma J. miR-378 serves as a prognostic biomarker in cholangiocarcinoma and promotes tumor proliferation, migration, and invasion[J]. Cancer Biomark, 2019, 24(2):173-181.
8. Zhou Z, Ma J. miR-378 serves as a prognostic biomarker in cholangiocarcinoma and promotes tumor proliferation, migration, and invasion[J]. Cancer Biomark, 2019, 24(2):173-181.
9. Zhou Z, Ma J. miR-378 serves as a prognostic biomarker in cholangiocarcinoma and promotes tumor proliferation, migration, and invasion[J]. Cancer Biomark, 2019, 24(2):173-181.
10. Zhou Z, Ma J. miR-378 serves as a prognostic biomarker in cholangiocarcinoma and promotes tumor proliferation, migration, and invasion[J]. Cancer Biomark, 2019, 24(2):173-181.
11. Chen, Zhang, Sun, et al. MicroRNA432 inhibits the aggressiveness of glioblastoma multiforme by directly targeting IGF-1R[J]. Int J Mol Med, 2020, 45(2):597-606.
12. Yang S, He H, Ma Q S, et al. Experimental study of the protective effects of SYVN1 against diabetic retinopathy[J]. Sci Rep, 2015, 5:14036.
13. Zhang J, Jiang S, Lu C, et al. SYVN1/GPX5 axis affects ischemia/reperfusion induced apoptosis of AC16 cells by regulating ROS generation[J]. Am J Transl Res, 2021, 13(5):4055-4067.
14. Ji F, Zhou M, Sun Z, et al. Integrative proteomics reveals the role of E3 ubiquitin ligase SYVN1 in hepatocellular carcinoma metastasis[J]. Cancer Commun (Lond), 2021, 41(10):1007-1023.
15. Yang CY, Wang J, Zhang JQ, et al. Human circular RNA hsa_circRNA_101705 (circTXNDC11) regulates renal cancer progression by regulating MAPK/ERK pathway. Bioengineered, 2021, 12(1): 4432-4441.
16. Zhang K, Fang T, Zhao D, et al. Circular RNA Circ_0008043 promotes the proliferation and metastasis of hepatocellular carcinoma cells by regulating the microRNA (miR)-326/RAB21 axis[J]. Bioengineered, 2022, 13(3):6600-6614.
17. Chen R X, Liu H L, Yang L L, et al. Circular RNA circRNA_0000285 promotes cervical cancer development by regulating FUS[J]. Eur Rev Med Pharmacol Sci, 2019, 23(20):8771-8778
18. Zhang J, Wang H, Wu K, et al. Dysregulated circRNA_100876 contributes to proliferation and metastasis of colorectal cancer by targeting microRNA-516b (miR-516b)[J]. Cancer Biol Ther, 2020, 21(8):733-740.
19. Gao, Jin, Zhang, et al. Silencing circRNA_001937 may inhibit cutaneous squamous cell carcinoma proliferation and induce apoptosis by preventing the sponging of the miRNA5973p/FOSL2 pathway[J]. Int J Mol Med, 2020, 46(5):1653-1660.
20. Wang X, Shi J, Niu Z, et al. MiR-216a-3p regulates the proliferation, apoptosis, migration, and invasion of lung cancer cells via targeting COPB2[J]. Biosci Biotechnol Biochem, 2020, 84(10):2014-2027.
21. Jin T, Zhang Y, Zhang T. MiR-524-5p Suppresses Migration, Invasion, and EMT Progression in Breast Cancer Cells Through Targeting FSTL1[J]. Cancer Biother Radiopharm, 2020, 35(10):789-801.
22. Qian XQ, Tang SS, Shen YM, et al. Notch1 Affects Chemo-resistance Through Regulating Epithelial-Mesenchymal Transition (EMT) in Epithelial Ovarian cancer cells[J]. Int J Med Sci, 2020, 17(9):1215-1223.
23. Yang G, Zhang Y, Yang J. Identification of Potentially Functional CircRNA-miRNA-mRNA Regulatory Network in Gastric Carcinoma using Bioinformatics Analysis[J]. Med Sci Monit, 2019, 25:8777-8796.
24. Wang Y, Xu W, Zu M, et al. Circular RNA circ_0021093 regulates miR-432/Annexin A2 pathway to promote hepatocellular carcinoma progression[J]. Anticancer Drugs, 2021, 32(5): 484-495.
25. Tong L, Yang H, Xiong W, et al. circ_100984-miR-432-3p axis regulated c-Jun/YBX-1/β-catenin feedback loop promotes bladder cancer progression[J]. Cancer Sci, 2021, 112(4): 1429-1442.
26. Fang Y, Yao Y, Mao K, et al. Circ_0132817 facilitates cell proliferation, migration, invasion and glycolysis by regulating the miR-432-5p/NOL4L axis in neuroblastoma[J]. Exp Brain Res, 2021, 239(6): 1841-1852.
27. Nan J, Chen W J, Zhang J W, et al. Downregulation of miR-432 activates Wnt/β-catenin signaling and promotes human hepatocellular carcinoma proliferation[J]. Oncotarget, 2015, 6(10):7866-79.
28. Gao Z, Wang H, Li H, et al. Long non-coding RNA CASC2 inhibits breast cancer cell growth and metastasis through the regulation of the miR-96-5p/SYVN1 pathway[J]. Int J Oncol, 2018, 53(5): 2081-2090.
29. Liu L, Yu L, Zeng C, et al. E3 Ubiquitin Ligase HRD1 Promotes Lung Tumorigenesis by Promoting Sirtuin 2 Ubiquitination and Degradation[J]. Mol Cell Biol, 2020, 40(7): e00257-19.
30. Tan X, He X, Fan Z. Upregulation of HRD1 promotes cell migration and invasion in colon cancer[J]. Mol Cell Biochem, 2019, 454(1-2): 1-9.
31. Cai Y, Jia Y. Circular RNA SOX5 promotes the proliferation and inhibits the apoptosis of the hepatocellular carcinoma cells by targeting miR-502-5p/synoviolin 1 axis[J]. Bioengineered, 2022, 13(2): 3362-3370.
32. Liu L, Long H, Wu Y, et al. HRD1-mediated PTEN degradation promotes cell proliferation and hepatocellular carcinoma progression[J]. Cell Signal, 2018, 50: 90-99.

**Figure legends**

**Figure 1. circ-0039459 levels in liver tumor cells and tissues**

A) Differentially expressed circRNA of liver cancer from the GEO dataset GSE155949. B) Levels of seven circRNAs in THLE-3, Huh7, and Hep3B cells were detected using qRT-PCR. C) circ-0039459 levels in liver cancer and paracancerous tissues were detected using qRT-PCR. D) Expression of linear RNA and circRNA after ribonuclease R treatment was detected using qRT-PCR. E) Expression of linear RNA and circRNA after actinomycin D treatment was detected using qRT-PCR. **P < 0.01.

**Figure 2.** **Effects of regulating circ-0039459 expression on apoptosis, migration, proliferation, invasion, and EMT of hepatocellular carcinoma cells**

A) circ-0039459 expression after si-circ-0039459 1#, si-circ-0039459 2#, and si-nc transfection into Hep3B and Huh7 cells was detected using qRT-PCR. B) Effects of interference with circ-0039459 expression detected using CCK-8 assay. C) Effects of interference with circ-0039459 expression were detected using flow cytometry. D) and E) Influence of interference with circ-0039459 expression on migration and invasion of liver cancer cells was detected using Transwell assay. F), G), H), and I) Effects of interference with circ-0039459 expression on E-cadherin, N-cadherin, and vimentin proteins in hepatocellular carcinoma cells were detected using qRT-PCR and western blot. ^*^P < 0.05, ^**^P < 0.01.

**Figure 3.** **Targeting relationship between circ-0039459 and miR-432**

A) circ-0039459 and miR-432 potential binding sites. B) Targeting relationship between circ-0039459 and miR-432 was verified using dual-luciferase reporter assay. C) Targeting relationship between circ-0039459 and miR-432 was verified using an RNA pull-down experiment. D) The influence on miR-432 expression was detected using qRT-PCR. E) miR-39459 expression in THLE-3, Hep3B, and Huh7 cells was detected using qRT-PCR. F) miR-432 expression in liver cancer and adjacent tissues was detected using qRT-PCR. ^**^P < 0.01, ^***^P < 0.001.

**Figure 4. Inhibition of miR-432 expression reverses the effects of interference with circ-0039459 expression on the proliferation, apoptosis, migration, invasion, and EMT of liver cancer cells**

A) miR-432 expression after the transfection of nc inhibitor, nc mimic, and mimic into Hep3B and Huh7 cells was detected using qRT-PCR. B) Proliferation level of hepatocellular carcinoma cells was detected using the CCK-8 assay. C) Apoptosis rate of hepatocellular carcinoma cells was detected using flow cytometry. D) and E) Migration and invasion levels of hepatocellular carcinoma cells were detected using the Transwell assay. F), G), H), and I) Expression levels of E-cadherin, N-cadherin, and vimentin proteins were detected using qRT-PCR and western blot. ^**^P < 0.01, ^#^P < 0.05, ^##^P < 0.01.

**Figure 5. Targeting relationship between miR-432 and SYVN1**

A) Potential binding site between miR-432 and SYVN1. B) Targeted relationship between miR-432 and SYVN1 was verified using a dual-luciferase reporter assay. C) Targeting relationship between miR-432 and SYVN1 was verified using an RNA pull-down experiment. D) The influence of interference with circ-0039459 and miR-432 expression on the expression of SYVN1 mRNA was detected using qRT-PCR. E) Expression of SYVN1 mRNA in THLE-3, Hep3B, and Huh7 cells was detected using qRT-PCR. F) Expression of SYVN1 mRNA in liver cancer and adjacent tissues was detected using qRT-PCR. ^* *^ P < 0.01, ^# #^ P < 0.01.

**Figure 6. Effect of SYVN1 overexpression reversing miR-432 overexpression on the proliferation, apoptosis, migration, invasion, and EMT of hepatocellular carcinoma cells**

A) Expression of SYVN1 after oe-nc and oe-SYVN1 transfection into Hep3B and Huh7 cells was detected using qRT-PCR. B) Proliferation level of hepatocellular carcinoma cells was detected using the CCK-8 assay. C) Apoptosis rate of hepatocellular carcinoma cells was detected using flow cytometry. D) and E) Migration and invasion levels of hepatocellular carcinoma cells were detected using the Transwell assay. F), G), H), and I) Expression level of E-cadherin, N-cadherin, and vimentin was detected using qRT-PCR and western blot. ^* *^P < 0.01, ^# #^ P < 0.01.
